# Single-motor and multi-motor motility properties of kinesin-6 family members

**DOI:** 10.1242/bio.059533

**Published:** 2022-10-14

**Authors:** Andrew Poulos, Breane G. Budaitis, Kristen J. Verhey

**Affiliations:** ^1^ Department of Cell and Developmental Biology, University of Michigan Medical School, Ann Arbor, MI 48109, USA; ^2^ Cellular and Molecular Biology Program, University of Michigan Medical School, Ann Arbor, MI 48109, USA

**Keywords:** Kinesin, Microtubule, Anaphase, KIF20A, KIF20B, KIF23

## Abstract

Kinesin motor proteins are responsible for orchestrating a variety of microtubule-based processes including intracellular transport, cell division, cytoskeletal organization, and cilium function. Members of the kinesin-6 family play critical roles in anaphase and cytokinesis during cell division as well as in cargo transport and microtubule organization during interphase, however little is known about their motility properties. We find that truncated versions of MKLP1 (*Hs*KIF23), MKLP2 (*Hs*KIF20A), and *Hs*KIF20B largely interact statically with microtubules as single molecules but can also undergo slow, processive motility, most prominently for MKLP2. In multi-motor assays, all kinesin-6 proteins were able to drive microtubule gliding and MKLP1 and KIF20B were also able to drive robust transport of both peroxisomes, a low-load cargo, and Golgi, a high-load cargo, in cells. In contrast, MKLP2 showed minimal transport of peroxisomes and was unable to drive Golgi dispersion. These results indicate that the three mammalian kinesin-6 motor proteins can undergo processive motility but differ in their ability to generate forces needed to drive cargo transport and microtubule organization in cells.

## Teaser

**Summary:** Kinesin-6 proteins can move processively along microtubules as single molecules and drive microtubule gliding in multi-motor teams. However, they differ in their ability to produce force for cargo transport in cells.

## INTRODUCTION

Kinesins are a superfamily of proteins responsible for orchestrating fundamental microtubule-based processes including cell division, intracellular trafficking, cytoskeletal organization, and cilium function (Verhey et al., 2011; Cross and McAinsh, 2014; Guillaud et al., 2020; Konjikusic et al., 2021). The kinesin-6 family consists of three subfamilies, two of which are conserved across eukaryotes, mitotic kinesin-like protein 1 (MKLP1: *Hs*KIF23, *Dm*Pavarotti, *Ce*ZEN-4) and mitotic kinesin-like protein 2 (MKLP2: *Hs*KIF20A, *Dm*Subito), whereas the third subfamily is vertebrate-specific, KIF20B [also known as mitotic phosphoprotein 1 (MPP1)].

The kinesin-6 family is best known for its roles in mitosis and cytokinesis in animal cells. MKLP1 and MKLP2 play important roles during anaphase in formation of the central spindle, a narrow region of antiparallel overlapping microtubules. MKLP1 is a component of the centralspindlin complex which promotes anti-parallel microtubule bundling during central spindle assembly (Nislow et al., 1992; Kuriyama et al., 1994; Jantsch-Plunger et al., 2000; Kuriyama et al., 2002; Mishima et al., 2002; Zhu et al., 2005; Pavicic-Kaltenbrunner et al., 2007) whereas MKLP2 is responsible for the transport of Chromosome Passenger Complex (CPC) components from the centromeres to the central spindle (Gruneberg et al., 2004; Neef et al., 2006; Cesario et al., 2006; Kitagawa et al., 2013; Landino et al., 2017; Serena et al., 2020; Adriaans et al., 2020). MKLP1 and MKLP2 also play critical roles during cytokinesis in the assembly and constriction of the contractile ring as inhibition of centralspindlin activity or MKLP2-driven delivery of the CPC leads to a reduction in constriction rate and increase in cytokinesis failure (Adams et al., 1998; Powers et al., 1998; Raich et al., 1998; Hill et al., 2000; Fontijn et al., 2001; Kuriyama et al., 2002; Matuliene and Kuriyama, 2002; Matuliene and Kuriyama, 2004; Guse et al., 2005; Yuce et al., 2005; Nishimura and Yonemura, 2006; Lewellyn et al., 2011; Lekomtsev et al., 2012; Kitagawa et al., 2013; Nguyen et al., 2014; Basant et al., 2015; Zhang and Glotzer, 2015; Lie-Jensen et al., 2019). Much less is known about the function of KIF20B, which regulates midbody maturation and is necessary for the completion of cytokinesis (Abaza et al., 2003; Kanehira et al., 2007; Janisch et al., 2018).

Kinesin-6 proteins also play important contributions in interphase of cycling cells as well as in post-mitotic cells. MKLP1 and *Dm*Pav are expressed during neuronal development and have been shown to regulate neurite outgrowth and dendrite formation by controlling microtubule organization (Sharp et al., 1997a,b; Yu et al., 1997; Ferhat et al., 1998; Yu et al., 2000; Lin et al., 2012; Del Castillo et al., 2015). MKLP2 was identified as a binding partner of Rab6 and functions with Rab6 in Golgi organization and/or transport of Golgi-to-ER vesicles (Echard et al., 1998; Majeed et al., 2014; Miserey-Lenkei et al., 2017; Hieda et al., 2021). KIF20B also plays an important role in neuronal development, particularly during corticogenesis. KIF20B was implicated in axonal transport of Shootin1 and loss of KIF20B function disrupts neurite outgrowth, polarization, and migration (Sapir et al., 2013; McNeely et al., 2017; Janisch et al., 2018).

**Fig. 1.**
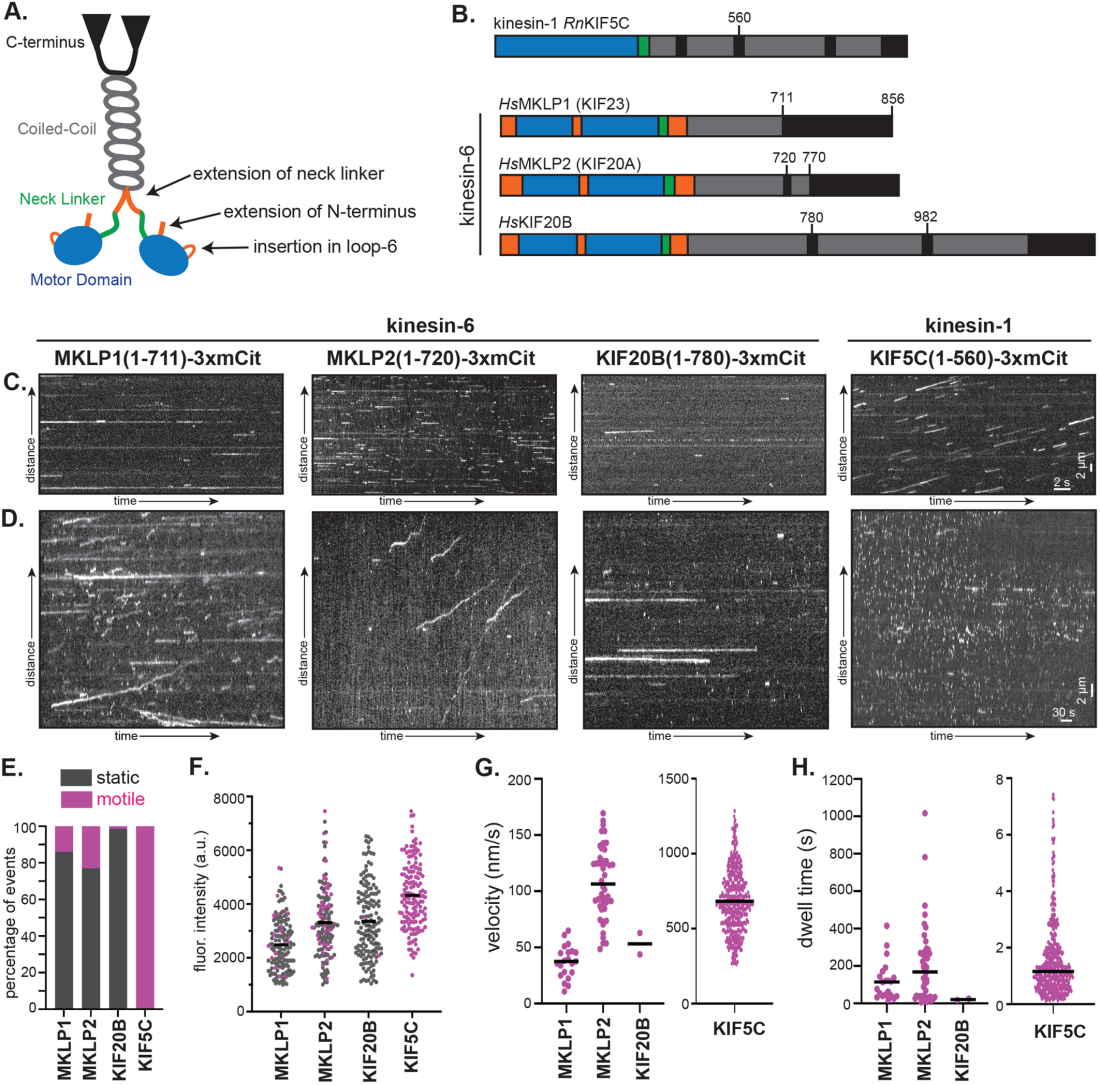
**Single-molecule motility properties of kinesin-6 motors.** (A) Schematic and (B) domain organization of kinesin-6 motor proteins. Blue, motor domain; green, neck linker; orange, insertions in kinesin-6 relative to kinesin-1; gray, predicted coiled coil. Numbers and black lines indicate the positions of protein truncations. (C-H) Motility properties of the indicated kinesin-6 proteins and the kinesin-1 control. All proteins were tagged at their C-terminus with three tandem mCitrine (3xmCit) fluorescent proteins. (C,D) Representative kymographs of imaging carried out using a (C) fast acquisition rate (1 frame every 50 ms, 30 s total) or (D) slow acquisition rate (1 frame every 2 s, 10 m total). Motility properties were determined from kymographs for kinesin-6 motors imaged at the slow acquisition rate and for kinesin-1 imaged at the fast acquisition rate. (E) The number of motile versus static events. (F) Fluorescence intensities of motile (magenta) and static (gray) motors measured from the first frame of the motor on the microtubule. *n*=150 events each. For the motile events, the (G) overall speed (black line, mean) and (H) dwell time (black line, median) were measured. Number of motile events: MKLP1(1-711)-3xmCit, *n*=21; MKLP2(1-720)-3xmCit, *n*=46; KIF20B(1-780)-3xmCit, *n*=2; KIF5C(1-560)-3xmCit, *n*=408 events across three independent experiments.

Kinesin proteins are defined by a highly conserved kinesin motor domain that contains signature sequences for nucleotide and microtubule binding. For most kinesins, nucleotide hydrolysis leads to unidirectional motility along the microtubule to drive transport of cargoes in cells. Other kinesins diffuse along or interact statically with microtubules. Understanding the motility properties of a kinesin protein is critical to understanding its functions in cells, yet the motility properties of members of the kinesin-6 family are largely unknown. The kinsesin-6 family is defined by three features of the motor domain: an N-terminal extension, an insertion in loop-6 on the surface of the motor domain, and an extended region between the neck linker and the first coiled coil predicted to drive homodimerization (Fig. 1A) (Mishima et al., 2002; Hizlan et al., 2006; Atherton et al., 2017; Guan et al., 2017; Janisch et al., 2018). The neck linker is important in force production and mechanochemical coordination of the two motor domains (Hwang et al., 2017; Hwang and Karplus, 2019) so the presence of an extended neck linker suggested that kinesin-6 proteins may not perform classical stepwise movement (Mishima et al., 2002; White et al., 2013; Davies et al., 2015; Atherton et al., 2017; Landino et al., 2017). Furthermore, MKLP1 and MKLP2 lack a key residue of the neck linker, the N-latch (Fig. S1A), that is required for force in generation in kinesin-1 (Budaitis et al., 2019), suggesting that these proteins may be impaired in their force generation.

The motility properties of individual kinesin proteins are typically examined in single-molecule motility assays where processive motors show unidirectional movement over time and non-processive motors show either diffusive or static binding along the microtubule lattice. However, an inability to undergo processive motility does not preclude a kinesin from driving microtubule-based transport events as non-processive kinesin proteins can work in teams drive motility (Furuta et al., 2013; Jonsson et al., 2015; Norris et al., 2018; Schimert et al., 2019). Single-molecule motility behavior of mammalian MKLP1 has not been evaluated, but multiple motors can drive the movement of microtubules in a gliding assay (Nislow et al., 1992). More progress has been made with the *C. elegans* homolog, *Ce*ZEN-4, which undergoes diffusive movement along the microtubule lattice as single motors, can undergo processive motility as clusters of motors, and can drive motility in microtubule gliding assays (Hutterer et al., 2009; Davies et al., 2015). *Ce*ZEN-4 and *Dm*Pav have also been observed to bundle microtubules in *in vitro* assays and have thus been suggested to function in the bundling of antiparallel microtubules at the central spindle and to resist microtubule sliding driven by kinesin-1 in neurons (Matuliene and Kuriyama, 2002; Mishima et al., 2002; Hutterer et al., 2009; Douglas et al., 2010; Del Castillo et al., 2015; Tao et al., 2016). For MKLP2, recent research showed that the full-length protein undergoes processive motility as single molecules and can transport purified CPC complexes along microtubules (Adriaans et al., 2020), suggesting that MKLP2 may be capable of a classic kinesin transport function. For *Hs*KIF20B, its single-molecule motility properties have not been described but it is capable of multi-motor transport in a microtubule gliding assay (Abaza et al., 2003). Finally, the sole member of the kinesin-6 family in fission yeast, *Sp*Klp9, forms homotetramers that display slow plus end-directed motility both as single molecules and in microtubule-gliding assays (Yukawa et al., 2019).

We utilized a variety of assays to characterize the single-molecule and multi-motor motility properties of kinesin-6 motors MKLP1, MKLP2, and KIF20B to understand their motility properties. We find that kinesin-6 motors largely interact statically with microtubules as single motors, with processive motility events primarily observed for MKLP1 and MKLP2. While all kinesin-6 motors can work in teams to drive transport in microtubule gliding assays, only MKLP1 and KIF20B can drive cargo transport in cells. Our results provide a basis for understanding kinesin-6 motility properties and can provide insight into how mutations in kinesin-6 motors can lead to disruption of neural development or cancer (Liu et al., 2014; Duan et al., 2016; McNeely et al., 2017).

## RESULTS

### Individual kinesin-6 proteins infrequently engage with microtubules for processive motility

We first tested whether kinesin-6 motors could undergo processive motility as individual motors using a standard single-molecule motility assay and total internal reflection fluorescence (TIRF) microscopy. Because many kinesin proteins utilize their C-terminal tail domains for autoinhibition of the N-terminal motor domain and/or as an auxiliary microtubule-binding domain (Verhey and Hammond, 2009; Tao et al., 2016; Zhang et al., 2020), we generated truncated versions of each kinesin-6 protein that contain the kinesin motor domain and a portion of the predicted coiled-coil segment for dimerization (Fig. 1B). For MKLP1 (*Hs*KIF23), we compared the truncated MKLP1(1-711) protein to the full-length MKLP1(1-856). For MKLP2 (*Hs*KIF23), we tested two truncated versions, MKLP2(1-720) and MLKP2(1-770) and for KIF20B, we also tested two truncated versions, KIF20B(1-780) and KIF20B(1-982). The proteins were tagged at their C-terminus with three tandem mCitrine (3xmCit) proteins for fluorescence imaging. In preliminary experiments, the shorter (Fig. 1C,D) and longer (Fig. S2B) versions of each protein behaved similar to each other. In addition, the shorter constructs behaved similarly when tagged at their C-terminus with either 3xmCit (Fig. 1C) or with tandem Halo and Flag tags (Fig. S2C). Thus, only the results from the 3xmCit-tagged shorter truncations will be described in detail.

We used single-molecule imaging to examine the ability of the truncated 3xmCit-tagged motors to undergo processive motility along taxol-stabilized microtubules. The well-characterized kinesin-1 protein KIF5C(1-560)-3xmCit (Cai et al., 2009) was used as control (Fig. 1B). Under standard imaging conditions (1 frame every 50 ms, 30 s total imaging), all kinesin-6 motor proteins were observed to transiently bind to and release from microtubules (Fig. 1C) and were occasionally observed to undergo directional motility at very slow velocities. We thus repeated the motility assays using a slower imaging rate (1 frame every 2 s) and a longer imaging time (15 min) to quantify the motility parameters. Most molecules again underwent transient binding to and release from the microtubules, however, processive, unidirectional events were observed for each kinesin-6 motor (Fig. 1D). Processive motility events were most frequently observed for MKLP2(1-720)-3xmCit (23% of events) whereas fewer processive events were observed for MKLP1(1-711)-3xmCit (14% of events) and KIF20B(1-780)-3xmCit (1.3% of events) (Fig. 1E, Table 1).

**Table 1.**
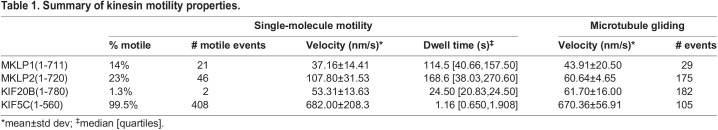
Summary of kinesin motility properties.

We tested whether each motor's oligomeric state (e.g. monomer or dimer) determined its ability to interact statically with (bind and release) or engage in motility along microtubules by measuring the fluorescence intensity of the protein at the first frame of its engagement with the microtubule. No correlation was found between fluorescence intensity and ability to undergo processive motility (Fig. 1F). The average fluorescence intensity of the kinesin-6 motors was lower than that of the control KIF5C(1-560)-3xmCit, a known dimer, but this may be due to the longer kinesin-6 motors being farther from the coverslip in the TIRF imaging field. We measured the properties of the motile events and found that MKLP1(1-711)-3xmCit moved with an average speed of 37.16±14.41 nm/s (mean±s.d.) and a median dwell time of 114.5 s [40.66, 157.50] (quartiles), MKLP2(1-720)-3xmCit moved with an average speed of 107.80±31.53 nm/s and a median dwell time of 168.6 s [38.03, 270.60], and KIF20B(1-780)-3xmCit moved with an average speed of 53.31±13.63 nm/s and a median dwell time of 24.50 s [20.83, 24.50] (Fig. 1G,H, Table 1). Taken together, these results show that kinesin-6 motors are capable of processive motility despite the presence of an extended neck linker.

### Kinesin-6 proteins can work in teams to drive microtubule gliding

**Fig. 2.**
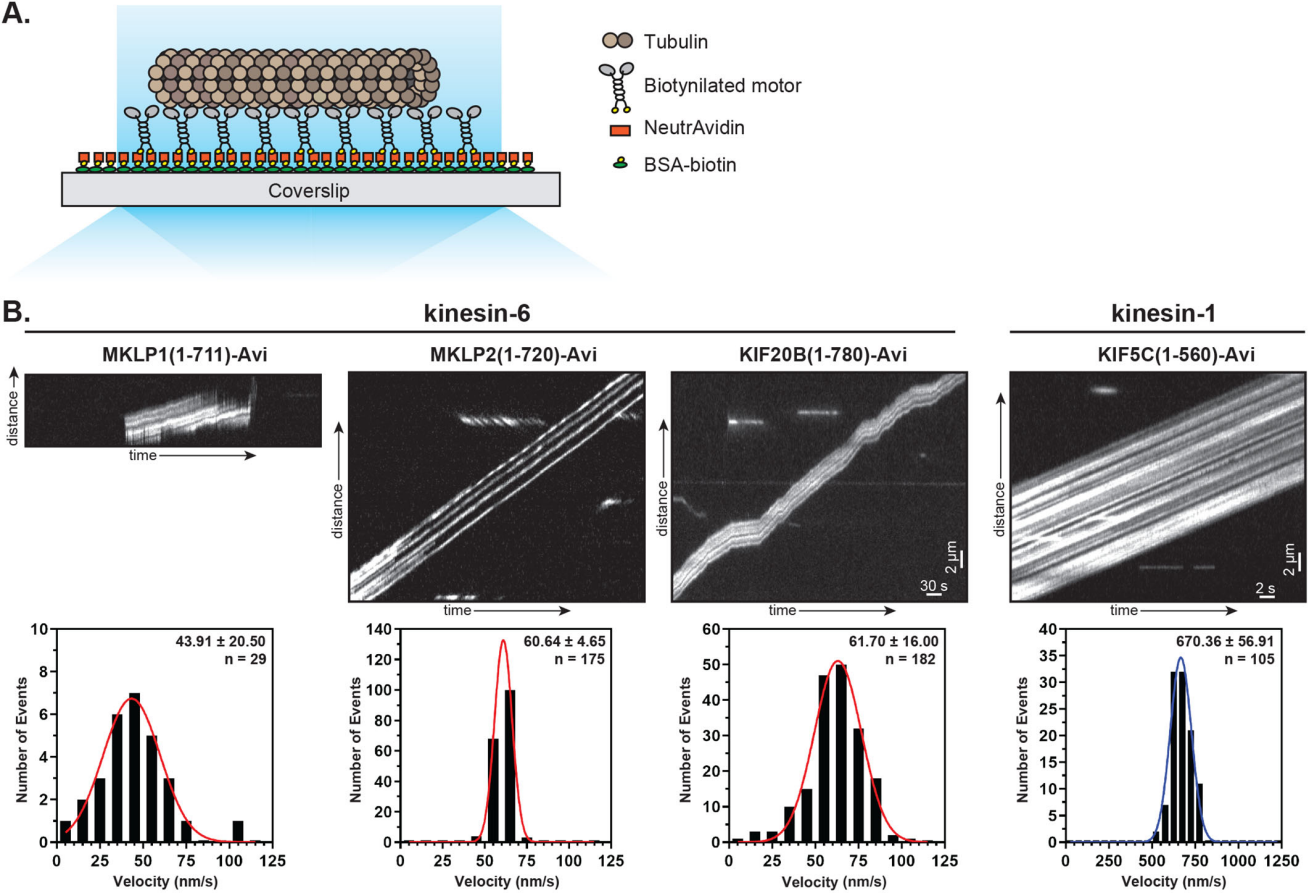
**Multi-motor properties of kinesin-6 motors in microtubule gliding assays.** (A) Schematic of the microtubule gliding assay. Biotinylated kinesin motors were bound to NeutrAvidin-coated coverslips and motor-driven microtubule gliding was imaged using TIRF microscopy. (B) Representative kymographs of fluorescent microtubules imaged at 1 frame/2 s and the kinesin-1 control imaged at 1 frame/100 ms. The velocity of microtubule gliding was calculated from kymographs and plotted as a histogram for the population. Insets display mean±s.d. and number of gliding events across four independent experiments for each construct.

We used a microtubule gliding assay to test whether kinesin-6 motors can work in teams to drive motility. To do this, the shorter kinesin-6 motor constructs were biotinylated by fusion of an AviTag to their C-terminus and co-expression with the enzyme BirA. The biotinylated motors were statically attached to a neutravidin-coated coverslip and then taxol-stabilized microtubules were introduced into the chamber (Fig. 2A). All three kinesin-6 motors were able to work in teams to glide microtubules (Fig. 2B). In each case, the speed of microtubule gliding was slow, similar to the speeds observed in single-molecule motility assays (Fig. 1), with average velocities of 43.91±20.50 nm/s (mean±s.d.) for MKLP1, 60.64±4.65 nm/s for MKLP2, and 61.70±16.00 nm/s for KIF20B (Fig. 2B, Table 1). These results indicate that although the kinesin-6 motor proteins infrequently engage with the microtubule for processive motility as single molecules, they can work in teams to drive microtubule gliding.

### MKLP1 and KIF20B persist at the midbody after completion of cytokinesis

**Fig. 3.**
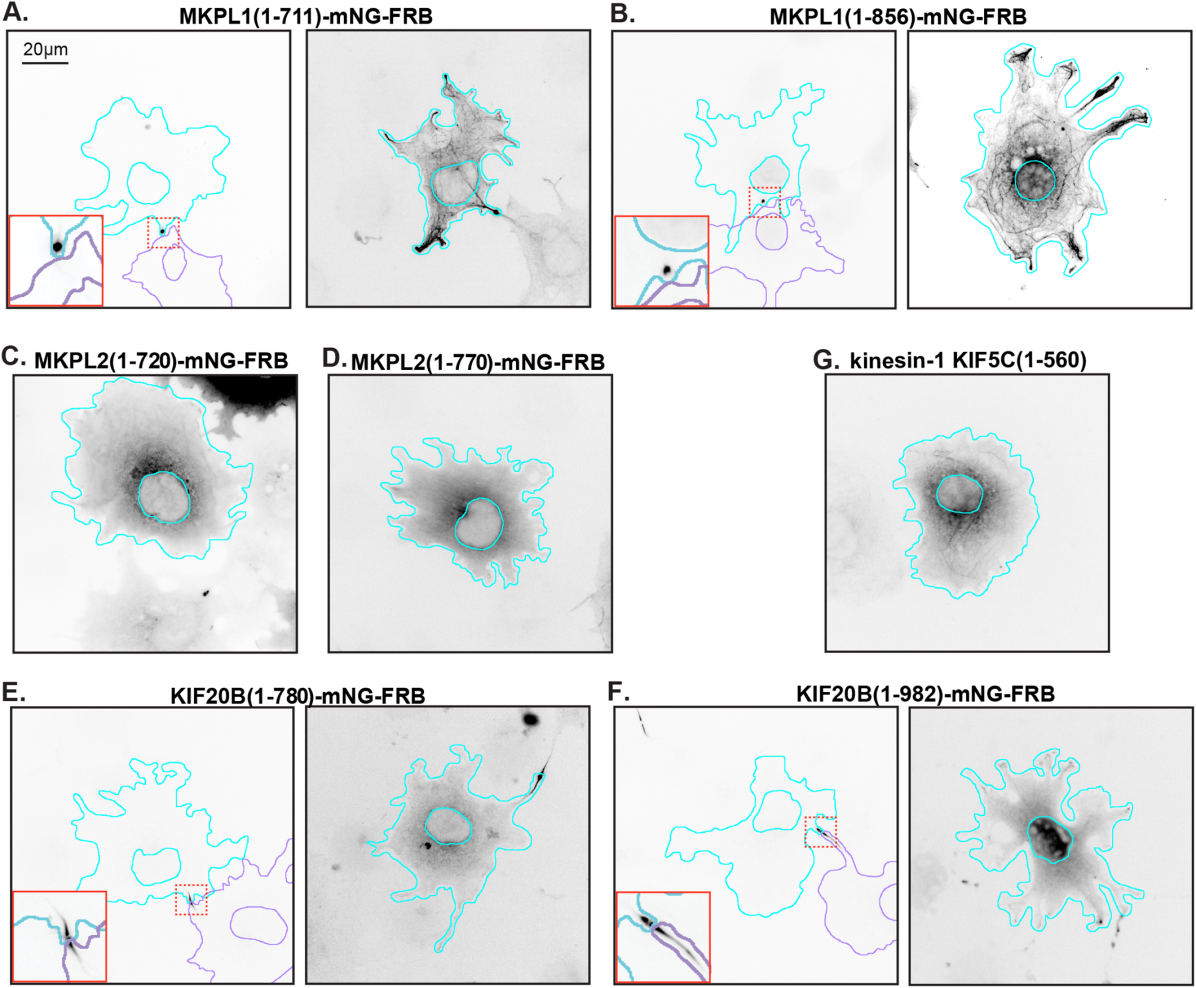
**Localization of kinesin-6 motor constructs in interphase cells.** Representative images of the localization of (A) truncated MKLP1(1-711) and (B) full-length MKLP1(1-856), (C) truncated MKLP2(1-720) and (D) MKLP2(1-770), (E) truncated KIF20B(1-780) and (F) KIF20B(1-982), and (G) kinesin-1 KIF5C(1-560) in COS-7 cells. All proteins were tagged at their C-terminus with three tandem mCitrine (3xmCit) fluorescent proteins. Images are displayed in inverted grayscale. Cyan lines and purple lines indicate the nucleus and periphery of a transfected cell. Red boxes in the lower left corner show a magnified view of the midbody region indicated by the boxes with dotted red lines. Scale bar: 20 μm.

To examine the kinesin-6 proteins in a cellular context, the proteins were tagged with monomeric NeonGreen (mNG) and FRB domains and expressed in COS-7 cells, whose large, flat morphology makes them preferred for fluorescence imaging. For MKLP1, the truncated MKLP1(1-711)-mNG-FRB and full-length MKLP1(1-856)-mNG-FRB proteins expressed only at low levels in the majority of cells and localized to the midbody of one daughter cell after cell division (Fig. 3A,B, red boxes in left panels). In cells with higher levels of expression, both MKLP1 versions localized along microtubules throughout the cytosol (Fig. 3A,B, right panels). For KIF20B, the truncated KIF20B(1-780)-mNG-FRB and KIF20B(1-982)-mNG-FRB proteins also expressed only at very low levels in the majority of cells and localized to the midbody, however, the KIF20B proteins were found on both sides of the midbody (Fig. 3E,F, red boxes in left panels). At higher levels of expression, the KIF20B proteins localized diffusely throughout the cell and not along interphase microtubules (Fig. 3E,F, right panels). For MKLP2, the truncated MKLP2(1-720)-mNG-FRB and MKLP(1-770)-mNG-FRB proteins localized diffusely throughout the cell, with faint localization to interphase microtubules in some cells, and did not persist at the midbody after completion of cell division (Fig. 3C,D).

### Kinesin-6 proteins can work in teams to drive cargo transport in cells

**Fig. 4.**
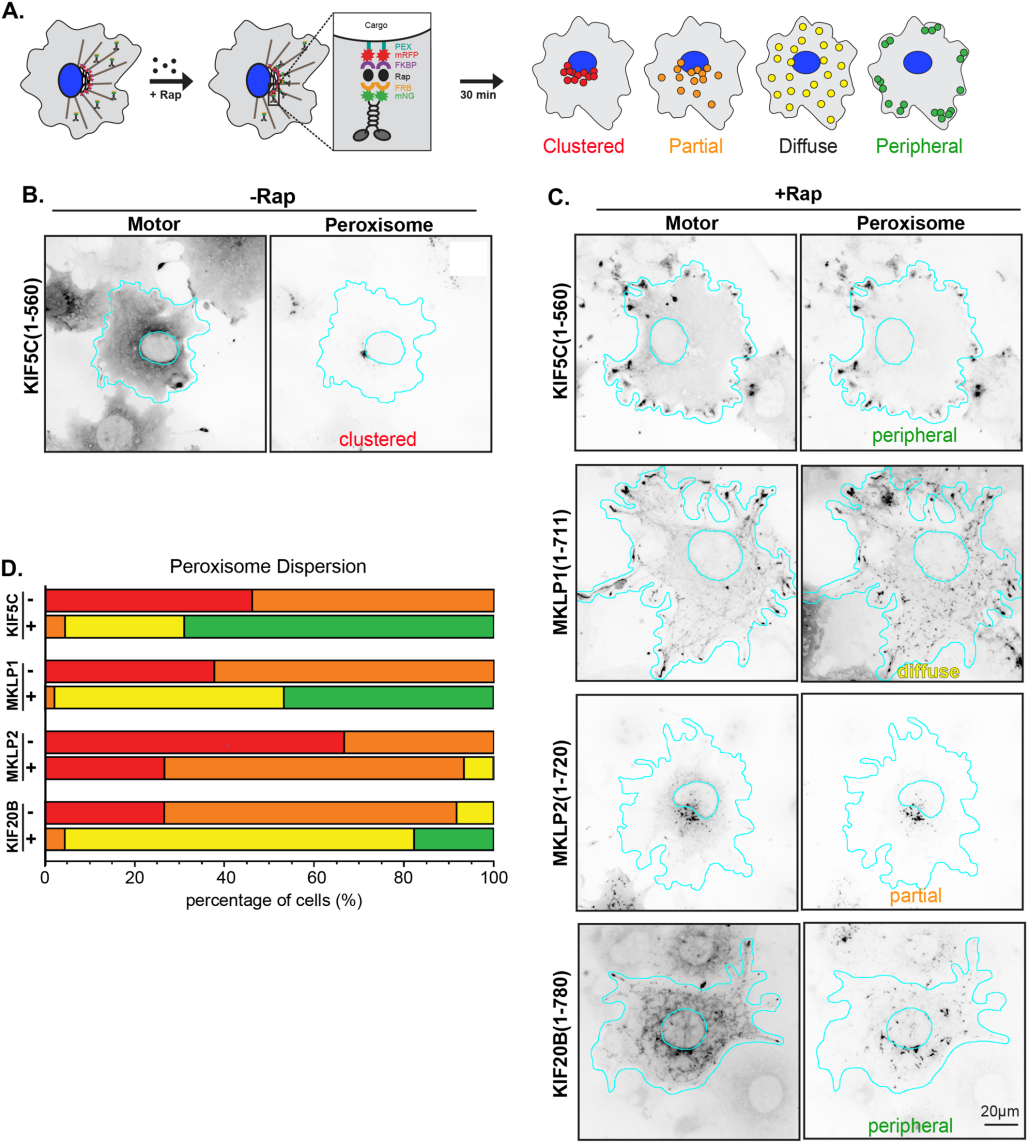
**Transport of low-load cargo (peroxisomes) by teams of kinesin-6 motors in cells.** (A) Schematic of the inducible cargo dispersion assay. COS-7 cells were co-transfected with plasmids encoding for the expression of a motor tagged with monomeric NeonGreen (mNG) and an FRB domain (motor-mNG-FRB) and a peroxisome-targeting sequence (PEX) tagged with monomeric red fluorescent protein (mRFP) and FKBP domain (CTS-mRFP-FKBP). Addition of rapamycin (+Rap) causes heterodimerization of the FRB and FKBP domains and recruitment of motors to the cargo membrane. Recruitment of active motors drives cargo dispersion to the cell periphery. Cells were fixed after 30 min and cargo dispersion was qualitatively scored as red, clustered; orange, partially dispersed; yellow, diffusely dispersed; or green, peripheral peroxisomes. (B,C) Representative images of motor-mNG-FRB and PEX-mRFP-FKBP protein localization (B) in the absence of Rap or (C) 30 min after addition of Rap. Images are displayed in inverted grayscale. Cyan lines indicate the nucleus and periphery of each cell. The scored phenotype is indicated at the bottom of the peroxisome image. Scale bar: 20 μm. (C) Peroxisome dispersion in individual cells was scored as indicated in A and the data for each construct are plotted as a stacked bar plot. *N*=45 cells from three independent experiments for each construct.

To test whether kinesin-6 proteins can work in teams to generate force, we utilized a cargo dispersion assay in which FRB-tagged motor proteins are recruited to FKBP-tagged organelles in a rapamycin-inducible manner and the resulting dispersion of that organelle is utilized as a measurement of the motor's ability to generate forces for cargo transport (Fig. 4A). Cargo dispersion assays have been widely used test the ability of kinesin motor proteins to drive transport (Kapitein et al., 2010; Efremov et al., 2014; Franker et al., 2016; Schimert et al., 2019; Vincent et al., 2020; Budaitis et al., 2019). We first tested whether the kinesin-6 motors could work in teams to drive dispersion of peroxisomes in cells. Peroxisomes localize to the perinuclear region of COS-7 cells and are relatively immotile under natural conditions (Rapp et al., 1996; Wiemer et al., 1997). We consider the peroxisome to be a low-load cargo, as it takes about 2-12 pN to move it from its natural location (Efremov et al., 2014). Motor-mNG-FRB constructs were co-expressed with a peroxisome-targeted FKBP protein tagged with mRFP (PEX-RFP-FKBP). The addition of rapamycin causes dimerization of the FRB and FKBP domains, resulting in targeting of the motor protein to the peroxisome (Fig. 4A). Peroxisome localization in the absence of and 30 min after addition of rapamycin was examined in fixed cells by fluorescence microscopy (Fig. 4B,C) and qualitatively scored as clustered (no motor-driven dispersion), partial dispersion, diffuse dispersion, and peripheral (complete peroxisome dispersion to the cell periphery) (Fig. 4A). We found that in cells where MKLP1- or KIF20B-mNG-FRB proteins localized to the midbody (Fig. 3A,C), the motor could not be recruited to the peroxisome surface upon addition of rapamycin. Thus, these cells were omitted from analysis, and cells were only scored for dispersion if the motor colocalized with peroxisomes after rapamycin treatment.

MKLP1(1-711)-mNG-FRB was able to drive robust peroxisome dispersion with 97.8% of cells showing peroxisomes with diffuse or peripheral localization (Fig. 4C,D), compared to 0% without rapamycin (Fig. 4D). This transport ability is comparable to that of the control kinesin-1 KIF5C(1-560) protein (Fig. 4B-D). Similarly, KIF20B(1-780)-mNG-FRB was also able to drive peroxisome dispersion as 95.6% of cells displayed diffuse or peripheral peroxisome localization after rapamycin (Fig. 4C,D), compared to 8.3% without rapamycin (Fig. 4D). However, MKLP2(1-720)-mNG-FRB showed a limited ability to drive peroxisome dispersion as the majority of cells (66.7%) showed only a partial dispersion (Fig. 4C,D).

**Fig. 5.**
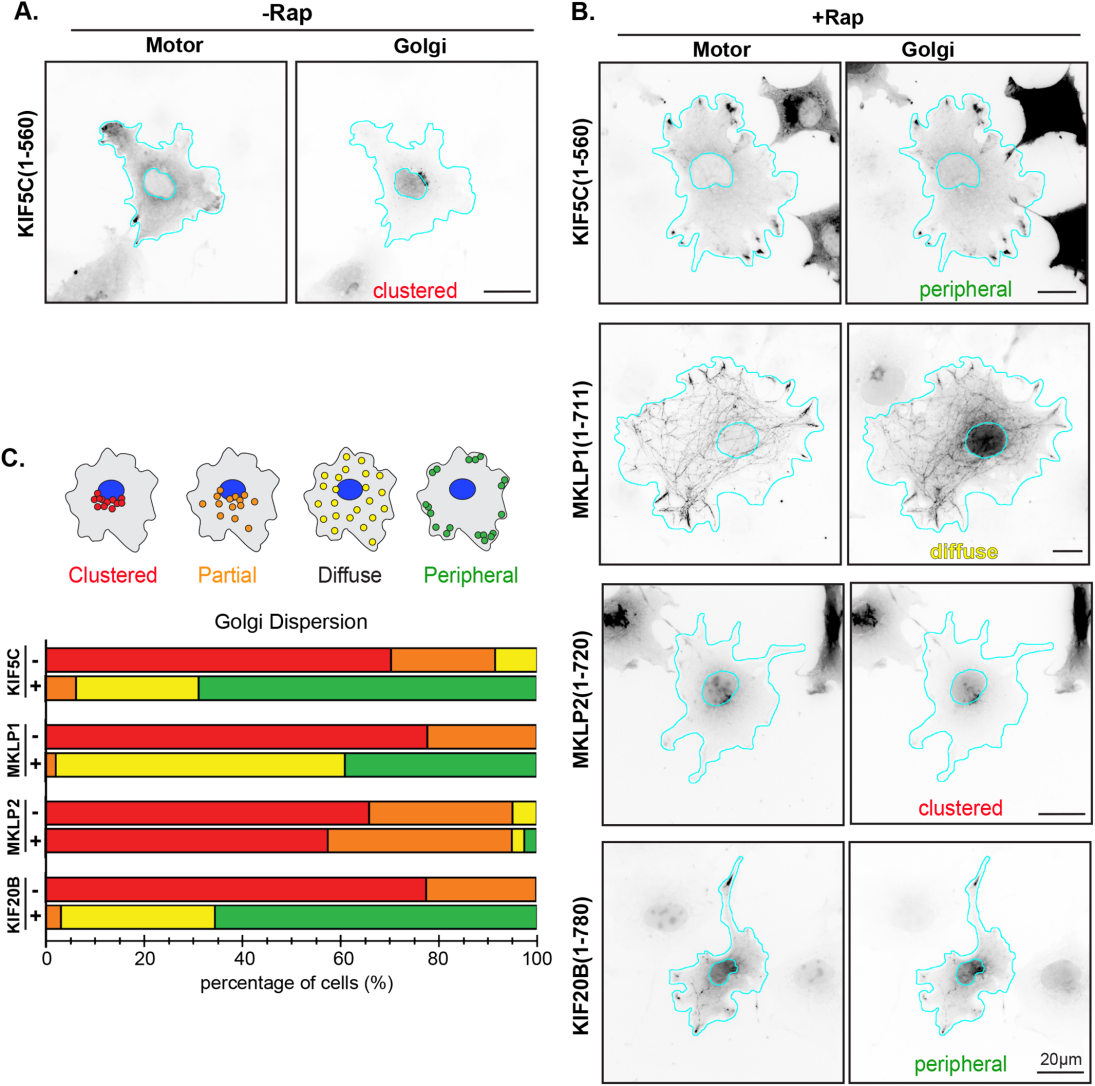
**Transport of high-load cargo (Golgi) by teams of kinesin-6 motors in cells.** (A,B) Representative images of motor-mNG-FRB and Golgi-mRFP-FKBP protein localization (A) in the absence of Rap or (B) 30 min after addition of Rap. Images are displayed in inverted grayscale. Cyan lines indicate the nucleus and periphery of each cell. The scored phenotype is indicated at the bottom of the peroxisome image. Scale bar: 20 μm. (C) Golgi localization in individual cells was scored as clustered (red), partially dispersed (orange), diffusely dispersed (yellow), or peripheral (green). The data for each construct are plotted as a stacked bar plot. *N*=45 cells from three independent experiments for each construct.

We then tested whether the kinesin-6 motors could work in teams to drive dispersion of the Golgi complex in cells. The Golgi complex is also localized to the perinuclear region of COS-7 cells and its localization is maintained by a variety of mechanisms including dynein-directed transport and tethering by myosin motors and linker proteins (Brownhill et al., 2009). We thus consider the Golgi to be a high-load cargo and recent research suggests that it takes ∼200 pN of force to be dispersed from its perinuclear position (Guet et al., 2014). Kinesin-6 motors tagged with mNG and FRB were coexpressed with a Golgi-targeted FKBP protein (GMAP210-RFP-FKBP). Golgi localization was examined in the absence of and 30 min after the addition of rapamycin and the same categorical analysis was used to quantify motor-driven dispersion of the Golgi as a cargo (Fig. 5C). MKLP1(1-711)-mNG-FRB was again able to drive robust transport of Golgi cargo, with 97.8% of cells displaying Golgi localized as either diffuse or peripheral (Fig. 5B,C) compared to 0% without rapamycin (Fig. 5C). KIF20B(1-780)-mNG-FRB also showed strong dispersion in this assay with 96.9% of cells displaying Golgi localized within the diffuse or peripheral categories (Fig. 5B,C), as compared to 0% without rapamycin (Fig. 5C). Thus, both MKLP1 and KIF2B show cargo transport properties comparable to that of the control kinesin-1 KIF5C(1-560) protein (Fig. 5A-C). Similar to what we observed in the peroxisome dispersion assay (Fig. 4), MKLP2(1-720)-mNG-FRB was unable to drive transport of high-load Golgi elements as cells expressing this motor showed a dispersion phenotype similar to that in the absence of rapamycin (Fig. 5B,C). Taken together, the cargo-dispersion assays suggest that MKLP1 and KIF20B, but not MKLP2, can work in teams to drive cargo transport in cells.

## DISCUSSION

### An extended neck linker does not prevent processive motility of mammalian kinesin-6 proteins

It was previously suggested that the presence of an extended region (∼60 aa) that separates the kinesin-6 neck linker and coiled-coil regions (Fig. 1A) would hinder coordination of the two motor domains required for processive motility (Mishima et al., 2002; White et al., 2013; Davies et al., 2015; Atherton et al., 2017; Landino et al., 2017). Indeed, other kinesin family members with extended neck linker domains have been found to be non-processive (Zhou et al., 2009; Terabayashi et al., 2012; He et al., 2014; Yue et al., 2018; Balseiro-Gómez et al., 2022). One exception to this model is recent work showing that the plant Phragmoplast-associated kinesin-related protein 2 (PAKRP2) exhibits processive unidirectional motility on microtubules as individual homodimers (Gicking et al., 2019). We find that while the majority of kinesin-6-microtubule interactions result in static binding, the motors are capable of processive motility albeit at slow speeds. Thus, while the extended neck linker does not prevent processive motility of kinesin-6 motors, it may limit their ability to convert a microtubule binding event into a processive motility event.

For MKLP1, our results are consistent with previous work showing that the majority of *Ce*ZEN-4(1-585) molecules attached briefly to microtubules without undergoing continuous movement (Hutterer et al., 2009). For *Ce*ZEN-4, a longer construct was shown to form clusters that undergo slow processive motility (Hutterer et al., 2009). We were unable to observe a similar clustering of our MKLP1 constructs, perhaps because the clustering element of *Ce*ZEN-4 (aa 585-601) is not conserved in *Hs*MKLP1 and/or our expression system lacks active Aurora B, which promotes MKLP1 clustering during anaphase (Douglas et al., 2010; Basant et al., 2015).

The ability of kinesin-6 motors to interact with microtubules and/or undergo processive motility is likely to be regulated by regions outside of the motor domain. Indeed, it was recently shown that full-length MKLP2-EGFP undergoes slow processive motility with no evidence of static interactions (Adriaans et al., 2020). It is thus possible that the C-terminal tail region may facilitate MKLP2's ability to convert microtubule binding to processive motility. Alternatively, the differences between our results and those of Adriaans et al. may reflect the use of 3xmCit versus EGFP tags and dynamic microtubules versus taxol-stabilized microtubules.

It is also possible that binding partners can modulate the microtubule interactions and/or motility of kinesin-6 proteins. For example, binding of its centralspindlin partner *Ce*CYK-4 (MgcRacGAP) results in an increase in the affinity of *Ce*ZEN-4 for microtubules but a decrease in velocity, which may play a role in centralspindlin's microtubule bundling activity (White et al., 2013; Davies et al., 2015). For MKLP2, binding of its anaphase cargo the CPC increases the ATPase activity and processivity of MKLP2 (Adriaans et al., 2020; Serena et al., 2020).

### Kinesin-6 motors can work in teams but only MKLP1 and KIF20B generate high forces

We find that all of the mammalian kinesin-6 motors can work in teams to drive microtubule gliding at slow speeds. These results are consistent with previous work using a gliding assay to investigate the motility of MKLP1 and its homologs *Ce*ZEN-4 and *Dm*Pav as well as KIF20B (Nislow et al., 1992; Abaza et al., 2003; Hutterer et al., 2009; Douglas et al., 2010; White et al., 2013; Davies et al., 2015; Tao et al., 2016). Interestingly, we find that kinesin-6 motors differ in their ability to drive cargo transport in cells. MKLP1 and KIF20B can work effectively in teams to drive the dispersion of both low-load and high-load organelles in cells, suggesting that these motors may be capable of high force output. In contrast, MKLP2 was only able to drive the limited dispersion of low-load peroxisomes in the same assay, suggesting that the force output of this motor may be impaired relative to kinesin-1.

Force generation by kinesin motors requires neck linker docking which occurs in two sequential steps: zippering of the neck linker with the coverstrand to form the cover-neck bundle (CNB) followed by latching of the neck linker to the surface of the motor domain via a conserved asparagine residue (N-latch) (Hwang et al., 2017; Hwang and Karplus, 2019). Of the kinesin-6 motors, KIF20B is the only member that contains the N-latch residue (Fig. S1A) and was thus predicted to be capable of high force production. In support of this prediction, we find that KIF20B can robustly drive transport of both low-load peroxisomes and high-load Golgi in cells. These results indicate that KIF20B can work in teams to drive robust cargo transport, consistent with its proposed functions in the cytokinetic furrow of mitotic cells and as a transport motor in developing neurons (Sapir et al., 2013; McNeely et al., 2017; Janisch et al., 2018).

The fact that MKLP1 and MKLP2 lack the N-latch residue (D in MKLP1 and Q in MKLP2, Fig. S1A) suggested that their force output may be lower than that of kinesin-1. Consistent with this hypothesis, we find that MKLP2 is unable to efficiently drive the transport of either low-load or high-load cargoes to the cell periphery. Interestingly, in addition to lacking the N-latch residue, the neck linker of MKLP2 contains more glycine and proline residues than other kinesins (Fig. S1A). These residues have a lower propensity for β-sheet formation (Fujiwara et al., 2012) and could thus also impair neck linker docking. Indeed, Atherton et al. were unable to resolve a docked neck linker conformation for MKLP2 even in the ATP- and microtubule-bound state (Atherton et al., 2017). Our finding that MKLP2 is only capable of low-load transport implies that transport of its CPC cargo during anaphase (Adriaans et al., 2020) does not require a high force output by the transporting motor.

In contrast, our results show that MKLP1 is capable of both low-load and high-load transport in cells. These results indicate that the presence of an N-latch residue is not absolutely required for force-dependent functions of kinesin proteins. It may be that the N-latch is required for robust force generation by individual kinesin proteins but not when working in teams. Alternatively, individual MKLP1 proteins may be capable of robust force generation due to unique sequences that compensate for the loss of the N-latch residue. Further work is needed to distinguish between these possibilities. High force generation by teams of MKLP1 proteins is likely critical for its functions in both cycling and post-mitotic cells, particularly in bunding and sliding anti-parallel microtubules in opposition to other motor- and microtubule-driven forces (Matuliene and Kuriyama, 2002; Mishima et al., 2002; Hutterer et al., 2009; Douglas et al., 2010; Del Castillo et al., 2015; Tao et al., 2016).

### MKLP1 and KIF20B differ in their midbody localization in interphase cells

Recent work suggests that kinesin-6 motors have functional roles in interphase of cycling cells as well as in post-mitotic cells. We find that in the majority of interphase cultured cells, truncated (1-711) and full-length (1-856) constructs of MKLP1 accumulate on one side of the midbody. The asymmetric distribution of MKLP1 is consistent with previous work showing that MKLP1 is a marker of the midbody ring remnant that is asymmetrically inherited by one daughter cell after completion of abscission (Mishima et al., 2002; Kuo et al., 2011; Isakson et al., 2013; Peterman et al., 2019) and can be detected extracellularly secreted midbody remnants (Rai et al., 2021). In cells with higher expression of MKLP1(1-711) and MKLP1(1-856), the proteins localized along the length of cytosolic microtubules, consistent with previous work analyzing the localization of MKLP1 tail-deletion mutants in interphase HEK293 cells (Zhang et al., 2020). In contrast to the asymmetric localization of MKLP1, the expressed KIF20B proteins associate with both sides of the midbody on the microtubule ‘arms’, consistent with previous work (Sapir et al., 2013; Janisch et al., 2018; Fritzler et al., 2019). In contrast, we did not observe MKLP2 at the midbody, consistent with its loss from the midbody after furrow ingression is complete (D'Avino and Capalbo, 2016).

## MATERIALS AND METHODS

### Plasmids

A truncated, constitutively active kinesin-1 [rat KIF5C(1-560)] was used as a control in all experiments (Cai et al., 2009). Plasmids contain cDNAs encoding the human kinesin-6 family members MKLP1 isoform 2 (*Hs*KIF23, Uniprot Q02241-2), MKLP2 isoform 1 (*Hs*KIF20A, Uniprot O95235), and *Hs*KIF20B Isoform 3 (Uniprot Q96Q89-3). The truncated versions MKLP1(1-711), MKLP2(1-720), and KIF20B(1-780) were generated by a combination of PCR, Gibson cloning, and gene synthesis. All plasmids were verified by DNA sequencing. MKLP1(1-711) lacks the sequences in exon 18 (Fig. S1B), which are present in KIF23 isoform 1 [also known as CHO1, Uniprot Q02241-1 (Kuriyama et al., 2002)] and thus likely reflects the core microtubule-based properties of both CHO1 and MKLP1 isoforms. KIF20B contained the protein sequence conflict E713K and natural variations N716I and H749L (Fig. S1C). Plasmids and sequences available upon request. Motors were tagged with three tandem monomeric Citrine fluorescent proteins (3xmCit) for single-molecule imaging assays (Cai et al., 2007), AviTag for gliding assays, or monomeric NeonGreen (mNG)-FRB for inducible cargo-dispersion assays in cells. MKLP1(1-711)-3xFLAG-Avi was cloned by inserting a dsDNA fragment encoding three tandem Flag tag into a digested MKLP1(1-711)-Avitag plasmid using Gibson assembly (HiFi DNA Assembly M5520, New England Biolabs). The peroxisome-targeting PEX3-mRFP-FKBP construct is described in (Kapitein et al., 2010) and the Golgi-targeting GMAP210p-mRFP-FKBP construct is described in (Engelke et al., 2016). Constructs coding for FRB (DmrA) and FKBP (DmrC) sequences were obtained from ARIAD Pharmaceuticals and are now available from Takara Bio Inc. Plasmids encoding monomeric NeonGreen were obtained from Allele Biotechnology.

### Cell culture, transfection, and lysate preparation

COS-7 (African green monkey kidney fibroblasts, American Type Culture Collection, RRID:CVCL_0224) were grown at 37°C with 5% (vol/vol) CO_2_ in Dulbecco's Modified Eagle Medium (Gibco) supplemented with 10% (vol/vol) Fetal Clone III (HyClone) and 2 mM GlutaMAX (L-alanyl-L-glutamine dipeptide in 0.85% NaCl, Gibco). Cells are checked annually for mycoplasma contamination and were authenticated through mass spectrometry (the protein sequences exactly match those in the African green monkey genome). Cells were seeded in 35 mm wells of a 6-well dish and transfected 24 h later with plasmids using TransIT-LT1 transfection reagent (Mirus) and Opti-MEM Reduced Serum Medium (Gibco). Cells were trypsinized and harvested 24 h after transfection by low-speed centrifugation at 3000×***g*** at 4°C for 3 min. The pellet was resuspended in cold 1X PBS, centrifuged at 3000×***g*** at 4°C for 3 min, and the pellet was resuspended in 50 μl of cold lysis buffer [25 mM HEPES/KOH, 115 mM potassium acetate, 5 mM sodium acetate, 5 mM MgCl_2_, 0.5 mM EGTA, and 1% (vol/vol) Triton X-100, pH 7.4] with 1 mM ATP, 1 mM phenylmethylsulfonyl fluoride, and 1% (vol/vol) protease inhibitor cocktail (P8340, Sigma-Aldrich). Lysates were clarified by centrifugation at 20,000×***g*** at 4°C for 10 min and lysates were snap frozen in liquid nitrogen and stored at −80°C.

### Single-molecule motility assays

Microtubules were polymerized (porcine tubulin unlabeled and HiLyte-647-labeled, Cytoskeleton Inc T240 and TL670) in BRB80 buffer [80 mM Pipes/KOH pH 6.8, 1 mM MgCl_2_, 1 mM EGTA] supplemented with GTP and MgCl_2_ and incubated for 60 min at 37°C. 20 μM taxol in prewarmed BRB80 was added and incubated for 60 min to stabilize microtubules. Microtubules were stored in the dark at room temperature for up to 2 weeks. Flow cells were prepared by attaching a #1.5 18 mm^2^ coverslip (Thermo Fisher Scientific) to a glass slide (Thermo Fisher Scientific) using double-sided tape. Microtubules were diluted in fresh BRB80 buffer supplemented with 20 μM taxol, infused into flow cells, and incubated for 5 min to allow for nonspecific absorption to the glass. Flow cells were then incubated with blocking buffer [5 mg/ml casein in P12 buffer supplemented with 5 μM taxol] for 5 min. Flow cells were then infused with motility mixture [0.5–5.0 μl of COS-7 cell lysate, 25 μl BRB80 buffer, 1 μl 100 mM ATP, 0.5 μl 100 mM MgCl_2,_ 0.5 μl 100 mM DTT, 0.5 μl 20 mg/ml glucose oxidase, 0.5 μl 8 mg/ml catalase, and 0.5 μl 1 M glucose], sealed with molten paraffin wax, and imaged on an inverted Nikon Ti-E/B TIRF microscope with a perfect focus system, a 100×1.49 NA oil immersion TIRF objective, three 20 mW diode lasers (488 nm, 561 nm, and 640 nm) and EMCCD camera (iXon^+^ DU879; Andor). Image acquisition was controlled using Nikon Elements software for 50 ms exposures at 1 frame/50 ms (30 s total imaging) or 1 frame/2 s (10 m total imaging) and all assays were performed at room temperature.

Motility data were analyzed by first generating maximum intensity projections to identify microtubule tracks (width=3 pixels) and then generating kymographs in Fiji (https://fiji.sc). Events that ended as a result of a motor reaching the end of a microtubule were included; therefore, the reported dwell times are likely an underestimation. The number of motile events obtained for each motor in each of the three independent experiments are: MKLP1(1-711)-3xmCit 4/39, 8/54, 9/57 (motile/total); for MKLP2(1-720)-3xmCit 12/58, 11/62, 17/80; for KIF20B(1-780)-3xmCit 0/41, 1/51, 1/58; for KIF5C(1-560)-3xmCit 108/109, 156/156, 144/145. For intensity measurements, a 9×9 pixel box was drawn around a fluorescent motor in the first frame that the motor was observed on a microtubule. 150 motility events from three experiments were randomly selected for each motor. Each event was noted as motile or static. The total pixel intensity inside the box was measured and the background intensity of a region lacking motors and microtubules was subtracted. Data were graphed using GraphPad Prism.

### Microtubule gliding assay

A flow cell was prepared, and microtubules were assembled as described in the single molecule section. Biotinylated motors were generated by coexpression of motors tagged with the 15-aa Avi tag and the bacterial biotin ligase BirA fused with HA tag (HA-BirA) in COS-7 cells. Biotinylated motors were attached to the coverslip surface by sequential incubation of flow cells with (A) 1 mg/ml BSA-biotin, (B) blocking buffer [0.5 mg/ml casein and 10 µM taxol in BRB80], (C) 0.5 mg/ml NeutrAvidin, (D) blocking buffer, and (E) blocking buffer containing cell lysates with 2 mM ATP, 10 mg/ml casein, 10 μM taxol. Taxol-stabilized HiLyte 647-labeled microtubules in motility mixture [2 mM ATP, 10 µM taxol, 2 mM MgCl_2_, and oxygen scavenging in BRB80] were then added, and the flow cells were sealed with molten paraffin wax and imaged by TIRF microscopy. For KIF5C, images were acquired continuously at 50 ms per frame for 30 s. For MKLP1, MKLP2, and KIF20B, images were acquired at one frame every 2 s for 10 min. Maximum-intensity projections were generated, and the kymographs were produced by drawing along these tracks (width=3 pixels) using Fiji (https://fiji.sc). Velocity was defined as the distance on the y axis of the kymograph divided by the time on the x axis of the kymograph.

For all three kinesin-6 motors, the amount of added cell lysate required to generate robust microtubule gliding was tested as addition of low amounts of cell lysate resulted in microtubule binding but not motility. For MKLP2 and KIF20B, 2 μl of cell lysate was sufficient for microtubule gliding. For MKLP1, the low level of MKLP1(1-711) expression required protein concentration from higher volumes of cell lysate. To do this, MKLP1(1-711)-Avi was further tagged with 3xFlag peptides [MKLP1(1-711)-3xFLAG-Avi]. Ten 10 cm plates of COS-7 cells were each co-transfected with 4.08 μg MKLP1(1-711)-3xFLAG-Avi and 4.08 μg HA-BirA plasmids using TransIT-LT1 transfection reagent (Mirus) and Opti-MEM Reduced Serum Medium (Gibco). Cells were trypsinized and harvested 24 h after transfection by low-speed centrifugation at 3000×***g*** at 4**°**C for 3 min. The pellet was resuspended in cold 1X PBS, centrifuged at 3000×***g*** at 4**°**C for 5 min, and the pellet was resuspended in 1000 μl of cold lysis buffer with 1 mM ATP, 1 mM phenylmethylsulfonyl fluoride, 1 mM DTT and 1% (vol/vol) protease inhibitor cocktail (P8340, Sigma-Aldrich). Insoluble material was removed by centrifugation at 20,000×***g*** at 4**°**C for 10 min. 50 μL anti-FLAG M2 beads (A2220, Sigma-Aldrich) was mixed into the supernatant for 1.5 h then washed twice with 2xFLAG wash buffer [300 mM KCl, 40 mM Imidazole/HCl, 10 mM MgCl_2_, 2 mM EDTA, and 2 mM EGTA] supplemented with 1 mM phenylmethylsulfonyl fluoride, 1 mM DTT, and 1% (vol/vol) protease inhibitor cocktail (P8340, Sigma-Aldrich). 3 mM ATP was added for the first wash only. Protein was eluted from the beads with 100 μl elution buffer [1% (vol/vol) BRB80, 1% (vol/vol) protease inhibitor cocktail (P8340, Sigma-Aldrich), 1 mM phenylmethylsulfonyl fluoride, 0.5 mM DTT, 0.1 mM ATP, and 0.5 mg/ml FLAG peptide (F4799, Sigma-Aldrich)]. The beads were pelleted by centrifugation at 20,000×***g*** at 4**°**C for 10 min and aliquots were snap frozen in liquid nitrogen and stored at −80**°**C.

### Inducible cargo dispersion assays

Plasmids for expression of kinesin-1 or kinesin-6 motors tagged with monomeric NeonGreen and an FRB domain were cotransfected into COS-7 cells with a plasmid for expression of PEX3-mRFP-FKBP or GMAP210p-mRFP-2xFKBP at a ratio of 5:1 with TransIT-LT1 transfection reagent (Mirus). Eighteen hours after transfection, rapamycin (Calbiochem, Sigma-Aldrich) was added to final concentration of 44 nM to promote FRB and FKBP heterodimerization and recruitment of motor the peroxisome or Golgi surface. Cells were fixed with 3.7% formaldehyde (Thermo Fisher Scientific) in 1X PBS, quenched in 50 mM ammonium chloride in PBS for 5 min, permeabilized for 5 min in 0.2% Triton-X 100 in PBS for 5 min, and blocked in 0.2% fish skin gelatin in PBS for 5 min. Primary and secondary antibodies were added to blocking buffer and incubated for 1 h at room temperature. Primary antibodies: polyclonal antibody against cis-Golgi marker giantin (1:1200 PRB-114C, Covance). Secondary antibodies: goat anti-rabbit Alexa680-labeled secondary antibody (1:500, Jackson ImmunoResearch). Cell nuclei were stained with 10.9 μM 4′,6-diamidino-2-phenylindole (DAPI, 1:1000 Sigma-Aldrich). Coverslips were mounted in ProlongGold (Invitrogen) and imaged using an inverted epifluorescence microscope (Nikon TE2000E) with a 40×0.75 NA objective and a CoolSnapHQ camera (Photometrics). Only cells expressing low levels of motor-mNG-FRB were scored (<4000 a.u.). Quantification of fluorescence intensities demonstrates that motor expression level does not correlate with dispersion phenotype (Fig. S3), as also noted by (Schimert et al., 2019). The corrected total cell fluorescence (CTCF) in the motor or peroxisome channel was quantified as CTCF=Integrated Density of Selected Cell – (Area of Selected Cell×Mean Fluorescence of Background) using Fiji (https://fiji.sc). Cargo localization before and after motor recruitment was scored as clustered, partial, diffuse, or peripheral dispersion based on the signal localization in the PEX3 (peroxisome) or giantin (Golgi) signal.

## Supplementary Material

10.1242/biolopen.059533_sup1 Supplementary information
