# Supplementary material for: Single-motor and multi-motor motility properties of kinesin-6 family members
Source: Biol Open. 2022 Oct 14;11(10):bio059533. doi: 10.1242/bio.059533 (PMC9581516; doi:10.1242/bio.059533)
Supplement: Supplementary information [file biolopen-11-059533-s1.pdf]

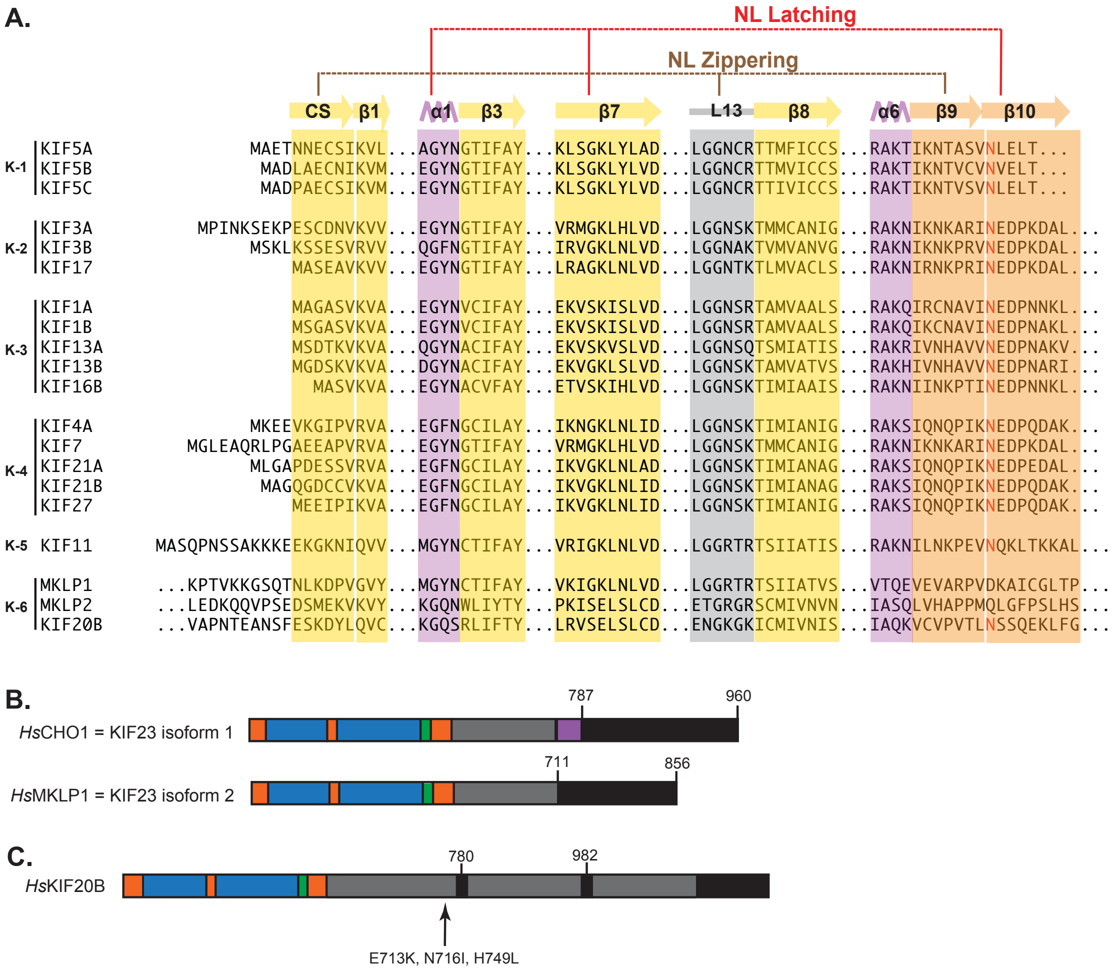

Fig. S1. Unique sequence features of human kinesin-6 motors.

(A) Alignment of sequences critical for neck linker docking in kinesin motor proteins. Neck linker docking occurs in two sequential steps. First, NL zippering which entails zippering of β9 of the neck linker with the coverstrand (CS) to form the cover-neck bundle (CNB). Second, NL latching which entails interactions of β10 of the neck linker with β7 and *α*1 regions and latching via a conserved asparagine residue (N-latch, red text). Yellow shading, alpha helices; Purple shading, beta sheets; Gray shading, loops.

(B) Comparison of the domain structures of KIF23 isoform 1 (CHO1) and isoform 2 (MKLP1). MKLP1 lacks the sequences of exon 18 (purple) which have been shown to be involved in actin binding (Kuriyama et al., 2002). (C) Location of sequence variants in *Hs*KIF20B. Uniprot entry Q96Q89-3 denotes E713K as a sequence conflict and N756I and H789L as natural variants. Using COILS, the presence of these variants is not predicated to alter the coiled coil nature of this region.

Biology Open • Supplementary information

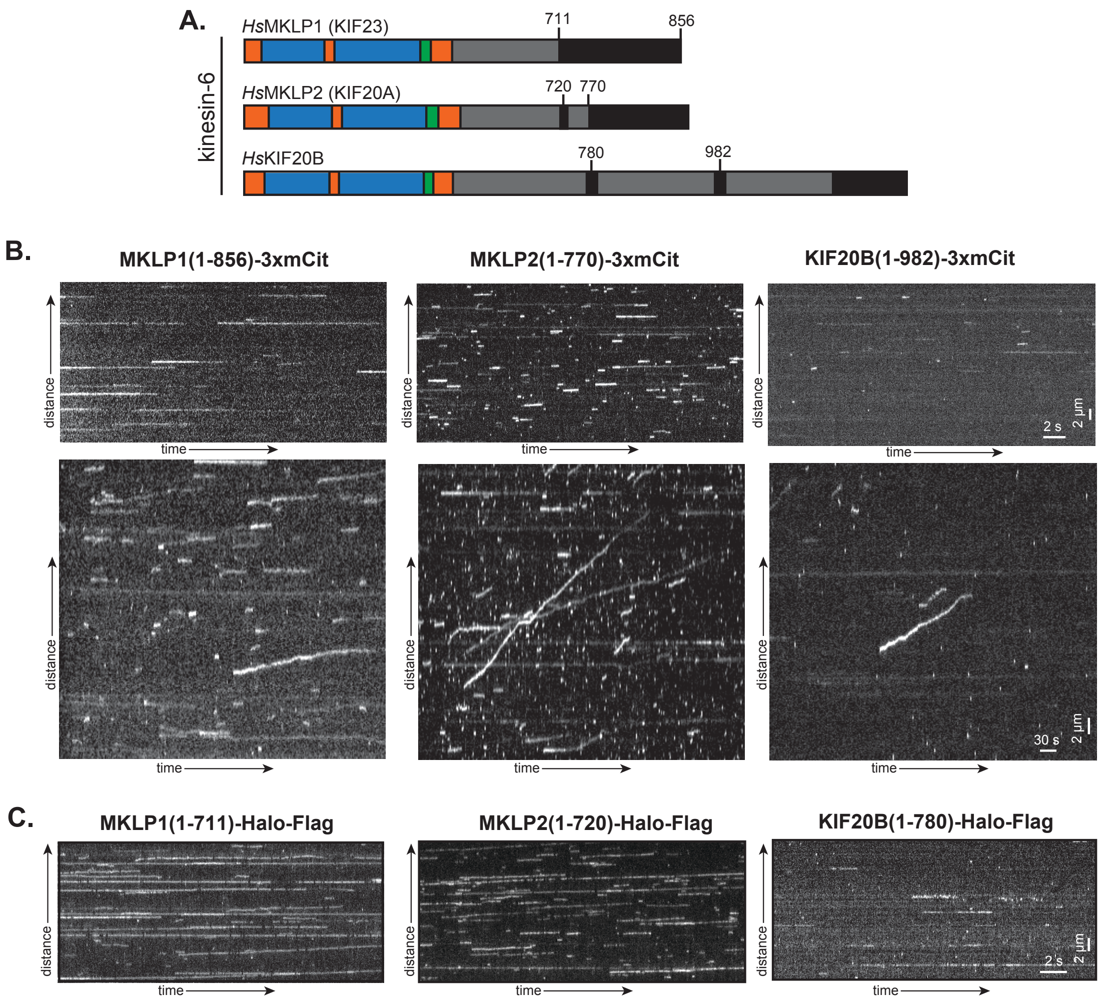

Fig. S2. Single-molecule imaging of additional kinesin-6 constructs.

(**A**) Domain organization and schematic of kinesin-6 motor proteins. Blue, motor domain; green, neck linker; orange, insertions in kinesin-6 compared to kinesin-1; gray, coiled coil. Numbers and black lines indicate the positions of protein truncations.

(**B**) Representative kymographs from TIRF imaging of longer kinesin-6 constructs. All proteins were tagged at their C-terminus with three tandem mCitrine (3xmCit) fluorescent proteins. Imaging was carried out a (top) fast acquisition rate (1 frame every 50 ms, 30 s total) or (bottom) slow acquisition rate (1 frame every 2 s, 10 m total). Time is on the x-axis and distance is on y-axis.

(**C**) Representative kymographs from TIRF imaging of shorter kinesin-6 constructs tagged at their C-terminus with tandem Halo and Flag tags. The proteins were labeled with JF552 Halo ligand. Imaging was carried out using a fast acquisition rate (1 frame every 50 ms, 30 s total).

Biology Open • Supplementary information

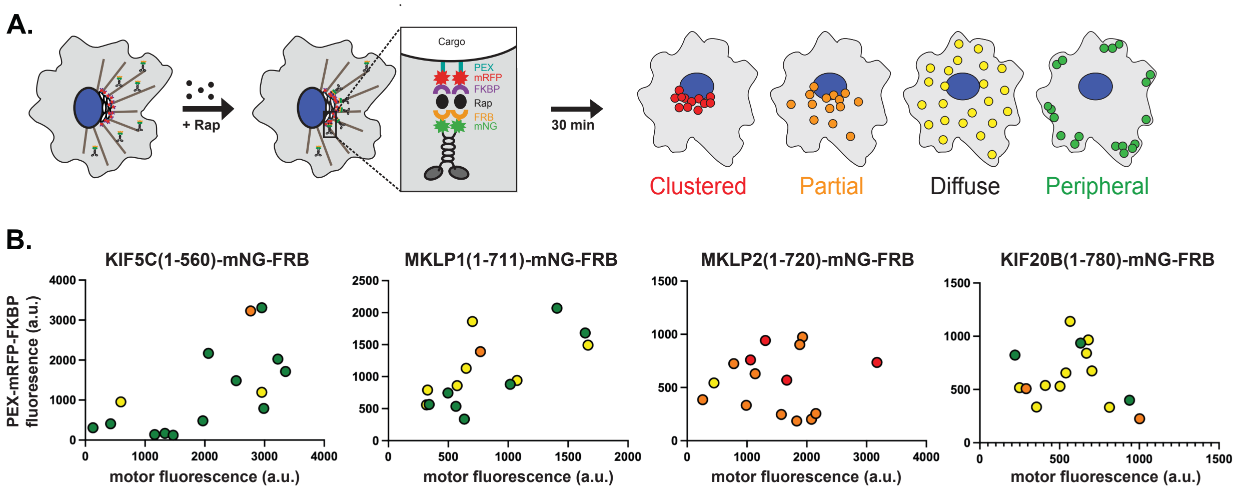

Fig. S3. Fluorescence intensities of expressed kinesin-6 motors in the inducible peroxisome dispersion assay. (**A**) Schematic of the assay. COS-7 cells were co-transfected with plasmids encoding for the expression of a motor tagged with monomeric NeonGreen (mNG) and an FRB domain (motor-mNG-FRB) and a peroxisome-targeting sequence (PEX) tagged with monomeric red fluorescent protein (mRFP) and FKBP domain.

(B) 15 cells were randomly selected for each construct and the whole cell fluorescence intensity was measured in the motor and peroxisome channels. Each spot represents one cell in which the peroxisome localization was scored as clustered (red), partially dispersed (orange), diffuse (yellow), or peripherally dispersed (green).

Biology Open • Supplementary information
